# Medial plantar nerve ligation as a novel model of neuropathic pain in mice: pharmacological and molecular characterization

**DOI:** 10.1038/srep26955

**Published:** 2016-05-27

**Authors:** Morena B. Sant’Anna, Ricardo Kusuda, Tiago A. Bozzo, Gabriel S. Bassi, José C. Alves-Filho, Fernando Q. Cunha, Sergio H. Ferreira, Guilherme R. Souza, Thiago M. Cunha

**Affiliations:** 1 Department of Pharmacology, Ribeirão Preto Medical School, University of São Paulo, Brazil; 2 Graduation Program in Basic and Apply Immunology, Ribeirão Preto Medical School, University of São Paulo, Ribeirão Preto, São Paulo, Brazil

## Abstract

Peripheral neuropathic pain is a consequence of an injury/disease of the peripheral nerves. The mechanisms involved in its pathophysiology are not entirely understood. To better understand the mechanisms involved in the development of peripheral nerve injury-induced neuropathic pain, more experimental models are required. Here, we developed a novel peripheral neuropathic pain model in mice by using a minimally invasive surgery and medial plantar nerve ligation (MPNL). After MPNL, mechanical allodynia was established, and mice quickly recovered from the surgery without any significant motor impairment. MPNL causes an increased expression of ATF-3 in the sensory neurons. At 14 days after surgery, gabapentin was capable of reversing the mechanical allodynia, whereas anti-inflammatory drugs and opioids were ineffective. MPNL-induced neuropathic pain was mediated by glial cells activation and the production of TNF-α and IL-6 in the spinal cord. These results indicate MPNL as a reasonable animal model for the study of peripheral neuropathic pain, presenting analgesic pharmacological predictivity to clinically used drugs. The results also showed molecular phenotypic changes similar to other peripheral neuropathic pain models, with the advantage of a lack of motor impairment. These features indicate that MPNL might be more appropriate for the study of neuropathic pain than classical models.

Neuropathic pain is widely recognized as one of the most difficult pain syndromes to treat and represents a significant challenge to researchers and clinicians. While it often does not respond to conventional analgesic therapies, non-conventional painkillers including anti-epileptics (such as carbamazepine and gabapentin) or antidepressants (such as amitriptyline) can be effective^1^. Nevertheless, patients continue to suffer with moderate severity pain despite taking prescribed medications for their condition^2^.

Neuropathic pain results from lesions in the central or peripheral nervous system caused by mechanical trauma, metabolic diseases, neurotoxic chemicals, infection, some medications or tumour invasion^3^. The identification of a specific pathophysiological change involved in the induction and maintenance of neuropathic pain may provide the basis for the development of novel analgesic therapies for this disease. In this context, the understanding of the neurobiology of neuropathic pain has been greatly accelerated by the development of animal models that reflect some elements of human pain syndromes^4^. Mechanical trauma in the peripheral nerves are the most used experimental models to induce neuropathic pain^5^. For instance, chronic constriction injury (CCI) caused by ligation of the sciatic nerve has been extensively used to elucidate the pathophysiology of peripheral neuropathic pain^6^,^7^,^8^,^9^,^10^. Importantly, these models require vast invasive surgeries, and in some cases, they result in damaged muscles or osteotomy. Consequently, animals require a long period to recover after surgery, which complicates the study of neuropathic pain in the initial period (induction phase). During this recovery phase, which lasts about three to five days, animals exhibit a considerable loss of functional gait, which makes the evaluation of pain behaviours difficult. Therefore, to better understand the mechanisms involved in the induction and maintenance of peripheral nerve injury-induced neuropathic pain, more experimental models are required. In the present study, we propose a novel model to study peripheral neuropathic pain that is based on a minimal invasive surgery and medial plantar nerve ligation (MPNL) in mice (Fig. 1). The pharmacological predictivity and molecular mechanisms involved in MPNL-induced peripheral neuropathic pain were also addressed.

## Results

### MPNL induced mechanical hypersensitivity and ATF-3 expression in the dorsal root ganglia

On the first day following the surgical procedure, the MPNL group showed a marked mechanical allodynia ipsilateral to the nerve injury, which was marginally different from sham-operated animals (Fig. 2A). However, MPNL-induced mechanical allodynia persisted, at least, twenty-five days after surgery, whereas the nociceptive threshold of sham-operated animals recovered to control levels (P < 0.001, n = 5; Fig. 2). In another set of experiments, we performed the MPNL model parallel to another well-characterized model of peripheral neuropathic pain in mice, the chronic constriction injury model (CCI). Both MPNL and CCI induced mechanical allodynia (Fig. 2B). However, the intensity of mechanical allodynia was higher in the CCI group compared with that in the MPNL group (Fig. 2B). Interestingly, in our conditions, it was only possible to assess the mechanical allodynia 7 days after the CCI surgery because some animals dragged their hind paw, which makes the mechanical threshold measurement difficult (Fig. 2B). Importantly, in the CCI model, 100% of the mice responded to the two last filaments (0.02 and 0.008 g) of the von Frey series, whereas none on the MPNL model mice reached this level of mechanical allodynia.

To evaluate whether the MPNL model would cause injury to peripheral sensory neurons, DRGs (L3–L6 pooled) were collected, and protein levels were assessed. There was a significant time-dependent increase in ATF-3 expression after MPNL, which peaked seven days post-injury, returning to control levels 21 days after MPNL (Fig. 3A). In another set of experiments, DRGs were harvested separately (L3 to L6; a poll of 3) at seven days following surgery to evaluate which DRG was more affected after MPNL by measuring ATF-3 expression. The increased expression was more prominent in L4 ganglia, although L3 and L5 also showed significantly higher ATF-3 expression compared with sham-control ganglia (Fig. 3B). Importantly, the up-regulation of ATF-3 was higher in the DRGs (L3–L6 pooled) of the CCI injured mice compared with the MPNL mice (Fig. 3C). Corroborating the western blotting data, the immunofluorescence of L4 ganglia revealed an increase in ATF-3 immunoreactivity in the nuclei of sensory neurons after MPNL compared with sham-operated DRGs, which was even higher in the CCI-operated mice (Fig. 4).

The motor functional capacity of animals undergoing MPNL surgery was evaluated using rota-rod test. No significant difference was observed between the sham and MPNL groups on the third (remaining time means of 117.6 and 119 min, respectively), seventh (means of 120 min in both groups) and fourteenth (means of 120 min in both groups) days after surgery. Corroborating the hypothesis that MPNL does not cause significant motor impairment, we found no cells expressing ATF-3 in the ventral horn of the spinal cord of the MPNL mice (Fig. 5). On the other hand, there was an increase in the ATF3-expressing cells in the ventral horn of the spinal cord of CCI mice, suggesting some degree of damage in the motor neurons (Fig. 5).

### Analgesic pharmacological predictive value of the MPNL model

The pharmacological predictive value of MPNL was determined by treating mice on the third and fourteenth days following MPNL with different classes of clinically used analgesic drugs, including steroids (dexamethasone; Fig. 6A,B), NSAIDs (indomethacin, Fig. 6C,D), opioids (morphine; Fig. 6E,F) and gabapentinoids (gabapentin, Fig. 6G,H). On the third day following MPNL, all drugs were significantly reversed mechanical allodynia (Fig. 6A,C,E,G). On the other hand, on the fourteenth day after surgery, dexamethasone, indomethacin and morphine produced only a slight reduction in mechanical allodynia after MPNL (Fig. 6B,D and F). Nevertheless, gabapentin reversed MPNL-induced mechanical allodynia by the 14th day after surgery (Fig. 6H).

### Glial cell activation in the spinal cord contributed to the MPNL-induced pain-like behaviours

Glial cell activation in the spinal cord (manly microglia and astrocytes) occurs after peripheral nerve injury and accounts for chronic pain induction and maintenance^11^,^12^,^13^,^14^. In this context, the participation of spinal glial cells in MPNL-induced pain-like behaviours was investigated. Firstly, microglial cell activation/proliferation in the spinal cord was assessed after MPNL induction through the analysis of Iba1 expression. There was a significant increase in the expression of *Aif1* (Iba1 gene) mRNA from the third to the tenth day after surgery (Fig. 7A). There was also a significant increase in Iba1 protein expression three days after MPNL (P < 0.01) (Fig. 7B). These results were confirmed by immunofluorescence of spinal cord slices. The naive group showed the constitutive expression of Iba1 in the spinal cord (Fig. 7C), whereas a massive increase in Iba1-expressing cells was observed on the ipsilateral side (dorsal horn) of the spinal cord 3 days after MPNL (Fig. 7C). In only 10% of the slices analysed, there was an increase in the expression of Iba1 in the ventral horn of the spinal cord (data not shown). Further supporting the hypothesis that MPNL triggers microglia activation in the spinal cord, we found that the morphology of microglia changed from a resting state (ramified) to an activated state (hypertrophied and amoeboid) after MPNL (Fig. 7C–F). Functionally, the inhibition of spinal microglia activity with intrathecal (i.t.) injections of minocycline (preoperatively, and once a day for up to 3 days following MPNL), significantly inhibited MPNL-induced mechanical allodynia in a dose- and time-dependent manner (Fig. 7G). The higher dose of minocycline did not change the mechanical threshold of sham-operated mice (Fig. 7G). Remarkably, when minocycline treatment was interrupted, the MPNL-induced mechanical allodynia was restored (Fig. 7G).

Regarding astrocytes, there was an increase in the expression of GFAP (astrocyte activation marker) in the spinal cord 7 days after MPNL (Fig. 8A). Because the increased expression of GFAP protein was significant on day 7 after surgery, this time point was used to assess the participation of astrocytes in MPNL-induced mechanical allodynia. Then, (i.t.) treatment with fluorocitrate (an astrocyte function inhibitor) reduced MPNL-induced mechanical allodynia in a dose- and time-dependent manner (Fig. 8B). Interestingly, the higher dose (3 nmol/site) of fluorocitrate reduced the mechanical allodynia until 24 h after MPNL, but the allodynia returned to the control levels 48 after treatment (Fig. 8B). The higher dose of fluorocitrate did not change the mechanical threshold of sham-operated mice (Fig. 8B).

### Role of spinal pro-inflammatory cytokines in the genesis of MPNL-induced pain-like behaviours

In the last part of this study, the involvement of pro-inflammatory cytokines (TNF and IL-6) in MPNL-induced mechanical allodynia was investigated. These two cytokines were chosen because they have been implicated in the genesis of neuropathic pain in other models^15^,^16^. Firstly, the mRNA expression of these cytokines was evaluated in the spinal cord following MPNL based on real-time PCR. There was a time-dependent increase in *Tnf* and *Il-6 gene* expression after surgery (Fig. 9A,B). Whereas *Tnf* expression peaked 1 and 3 days after MPNL and returned to control levels 5 days after surgery, *Il-6* expression was increased from day 3 until day 7, decreasing thereafter (Fig. 9A,B). Supporting these data, TNF and IL-6 protein expression was also up regulated in the spinal cord of MPNL mice at 3 days after surgery (Fig. 9C,D). To investigate whether these cytokines are involved in the genesis of MPNL-induced pain-like behaviours, MPNL was performed in C57BL/6 (WT), TNFR1/R2^−/−^ and IL-6^−/−^ mice. Mechanical allodynia triggered by MPNL was completely abrogated in TNFR1/R2^−/−^ mice (Fig. 9E). As further support, the i.t. injection of infliximab (anti-TNF antibody) also reduced MPNL-induced mechanical allodynia (Fig. 9F). In addition, mechanical allodynia induced by MPNL was only slightly reduced in IL-6^−/−^ compared with WT mice (Fig. 9G).

## Discussion

Neuropathic pain is a multidimensional chronic pain condition that is frequently caused by peripheral or central nerve injury or disease. These damaged nerves continuously send incorrect signals to the spinal cord and supraspinal sites, triggering changes in the structure, function and organization within the nervous system. One important cause of peripheral neuropathic pain is physical trauma to the peripheral nerves. However, the cellular and molecular mechanisms involved in peripheral neuropathic pain are not entirely understood. To better understand the mechanisms involved in peripheral neuropathic pain, in the present study, we developed a novel mouse model of this condition by using MPNL. To our knowledge, this is the most distal model of peripheral nerve injury described in mice that leads to a phenotype of pain hypersensitivity that might resemble human peripheral neuropathic pain syndromes.

One important sensory characteristic/symptom of neuropathic pain conditions is the development of mechanical hypersensitivity, which is clinically described as tactile allodynia^17^. In this context, it was clearly observed that after MPNL, animals developed hypersensitivity to mechanical stimulation. Notably, mechanical hypersensitivity was not accompanied by any significant motor impairment even at the earlier time points (one day after surgery). This might be an advantage compared with other models of peripheral neuropathic pain in which motor deficits can influence the determination of the nociceptive threshold^10^,^18^. This hypothesis is supported by our present findings in which the MPNL model did not damage motor neurons in the ventral horn of the spinal cord, whereas CCI did. In fact, the medial plantar nerve innervates the medial plantar region of the animal paw, which does not compromise the muscles responsible for the paw withdrawal reflex. Furthermore, the surgery performed to produce MPNL requires a tiny incision and did not involve any injury to muscles that are important for mouse gait. In addition, autotomy was not observed after MPNL. Signals of neuropathic pain without significant motor neuron damage and motor impairment has been also described in other models of peripheral nerve injury in rats^19^,^20^,^21^. Another important characteristic of the present model of neuropathic pain is the intensity of mechanical hypersensitivity developed after MPNL. In contrast with other models of peripheral neuropathic pain, such as CCI (Fig. 2), MPNL did not induce a maximal response in the von Frey test, allowing the study of mechanisms or drugs that perhaps potentiate this symptom. Finally, from the point of view of the experimenter, MPNL is very easy and fast (3–5 min) to perform.

The current understanding of neuropathic pain implies that the pain develops because of neuronal injury or diseases^22^,^23^. To access whether the MPNL model induces neuronal damage, the expression of ATF3 was analysed. ATF3 is a member of the ATF/CREB family of basic leucine zipper (bZip) transcription factors^24^. It is not constitutively expressed^25^. However, ATF3 expression can be induced in various tissues undergoing stress or injury including sensory neurons. Indeed, its expression in the cell body of primary sensory neurons has been used as an indirect marker of neuronal injury^26^,^27^,^28^. In fact, after a physical injury to the peripheral nerves in several models of neuropathic pain, ATF3 is upregulated^27^,^28^. MPNL induces an increase in ATF3 expression in the DRGs, supporting the hypothesis that MPNL might be a real model of neuropathic pain. Interestingly, it seems that the most affected neurons in the MPNL model are located in L4 DRG. In addition, it is important to mention that compared with CCI, MPNL is less severe in terms of promoting damage to the sensory neurons.

An important feature of a novel model for human diseases is the pharmacological predictivity. The MPNL model presents the predictive value to clinical drugs used for the treatment of neuropathic pain. Interestingly, MPNL-induced mechanical hypersensitivity was sensitive to classical anti-inflammatory drugs (NSAIDs) and opioids (morphine) at an earlier time point (3 days after surgery), suggesting an important inflammatory component in the induction phase. However, at later time points, only gabapentin exhibited a significant anti-nociceptive effect, which is similar to that observed when the neuropathic pain develops in human patients^29^,^30^. In fact, NSAIDs and steroidal anti-inflammatory drugs are poorly effective or even ineffective at alleviating neuropathic pain in humans or animal models of neuropathic pain^31^,^32^. In addition, in a recent systematic review and meta-analysis of pharmacological treatment of neuropathic pain, gabapentin was shown to be one of the first-line drugs^29^. These results indicate that MPNL is a predictive model for drugs that could act against neuropathic pain.

Neuropathic pain is a result of neuroplasticity across the nociceptive system^33^,^34^,^35^,^36^. Both peripheral and central sensitization are observed^37^,^38^. In addition to intrinsic neural mechanisms, there is a growing recognition of the importance of non-neuronal cells, especially glial cells, in the genesis and maintenance of chronic pain^39^ suggesting that these cells may be a new target for drug development^3^,^40^,^41^. Supporting this hypothesis is the finding that after MPNL surgery, there is a significant activation of microglial cells and astrocytes at the level of the spinal cord. The relevance of glial cell activation in MPNL-induced neuropathic pain behaviours was confirmed with the treatment of mice with glial cell inhibitors, as demonstrated in other models of neuropathic pain, such as spared nerve injury (SNI) and chronic constriction injury (CCI) models^42^,^43^. The mechanisms by which spinal glial cells mediate neuropathic pain seem to be dependent on their ability to produce cytokines, such as TNF and IL-6^44^,^45^,^46^. The expression of these cytokines is increased in the spinal cord after peripheral nerve injury^47^,^48^,^49^. Moreover, they are intimately involved in the maintenance of neuropathic pain by altering nociceptive transmission in the spinal cord^15^,^16^,^50^. Similar results were obtained in the spinal cord of mice that underwent MPNL, which presented a time-dependent increase in the expression of TNF and IL-6. Interestingly, TNFR1/2^−/−^ mice are totally protected from MPNL-induced mechanical hypersensitivity, suggesting that TNF plays an essential role in the induction and maintenance of MPLN-induced neuropathic pain. Although these results indicate that the spinal production of TNF mediates MPNL-induced neuropathic pain-like behaviours, using fully deficient mice, we cannot exclude that the TNF produced at the level of neuronal injury might play a role. In fact, there is an increase in the TNF levels at the site of peripheral nerve injury^51^,^52^. Supporting that spinal TNF mediates MPNL-induced pain hypersensitivity, intrathecal anti-TNF antibody treatment reduced this symptom. Strikingly, the efficacy of anti-TNF antibody i.t. treatment was reduced 24 h after the end of the treatment. This effect is likely due to the pharmacokinetics of the antibody when it is injected through the intrathecal route because it could diffuse through the cerebrospinal fluid. Regarding IL-6, it seems to be less relevant for neuropathic pain development after MPNL. Notably, although TNF and IL-6 were used to validate the molecular mechanisms involved in MPNL-induced neuropathic pain, we cannot rule out the participation of the other cytokines in this proposed model^53^.

In summary, we have shown that the MPNL model has features of peripheral neuropathy that mimic carpal/tarsal tunnel painful syndrome. Furthermore, we emphasize its advantages because it requires less invasive and complex surgery to induce, produces milder mechanical hypersensitivity, does not cause motor impairment, and mice have a quick recovery after surgery. Furthermore, its present pharmacological predictive value to analgesics is clinically useful. Finally, the proposed model has pathophysiological characteristics (molecular and cellular) that are similar to other peripheral neuropathic pain models. In conclusion, we suggest that MPNL might be a very suitable model for understanding the pathophysiological mechanisms of peripheral neuropathic pain and for testing novel analgesic compounds.

## Materials and Methods

### Animals

The experiments were performed in C57BL/6 male mice (wild type, WT, 20–25 g) and mice deficient ^(−/−)^ for the following proteins: TNF receptor type 1 and 2 (TNFR1/2^−/−^) and IL-6^−/−^. All animals were housed in the animal care facility of Ribeirao Preto Medical School, University of Sao Paulo. Animals were taken to the testing room at least 1 hour before the experiments and were used only once. Food and water were available ad libitum. The animal care and handling procedures were in accordance with the International Association for the Study of Pain guidelines for those animals used in pain research, and they were approved by the Committee for Ethics in Animal Research of the Ribeirao Preto Medical School-USP (Process n° 128/2011).

### Neuropathic pain models

#### MPNL model

**Figure 1:**
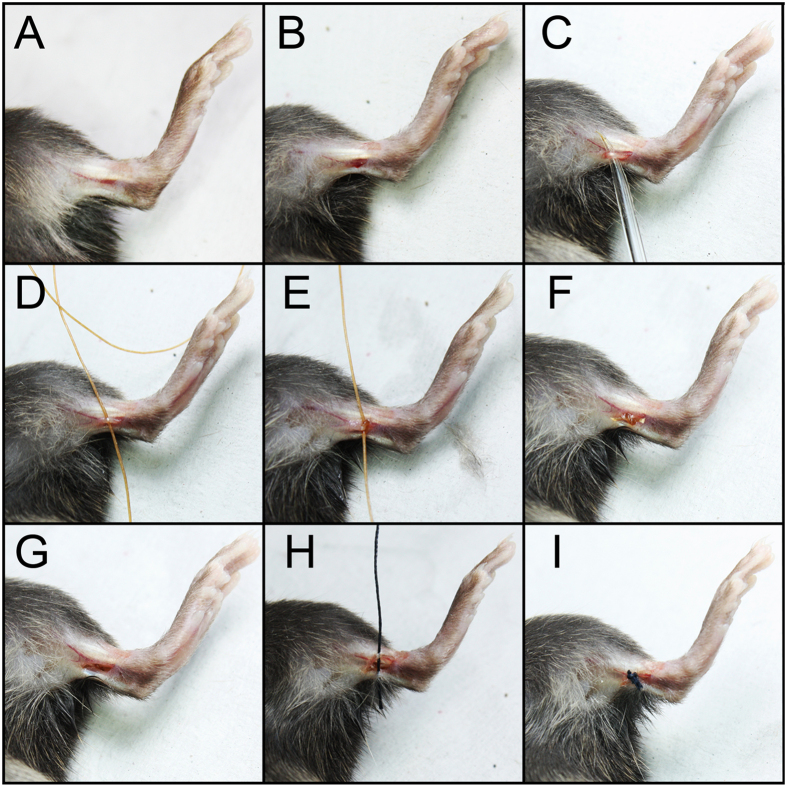
Representative image of the steps involved in the induction of MPNL. (**A**) Mice under anaesthesia and medial paw region, site of procedure; (**B**) incisions of approximately 0.5 cm; (**C**) MPN exposure with a glass probe; (**D–G**) MPN ligation (**H,I**) suture of the incision.

**Figure 2:**
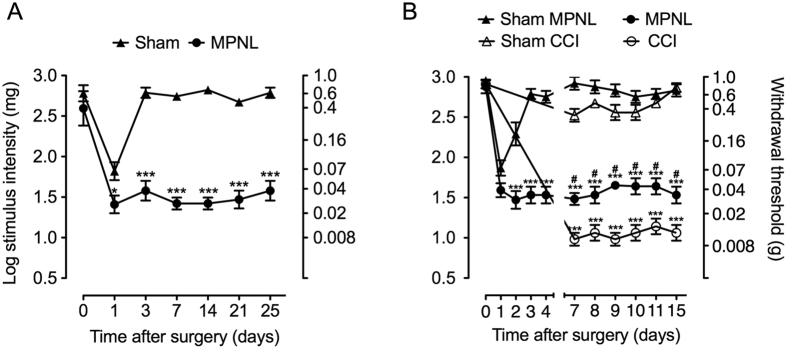
Mice developed mechanical hypersensitivity after MPNL. **(A)** The mechanical withdrawal threshold was evaluated using von Frey filaments before and from the 1st to 25th day after surgery. (**B**) The mechanical withdrawal threshold was evaluated before and at indicated time points after MPNL or CCI surgery. Sham-operated mice were used as the control. The data are presented as the mean ± S.E.M (n = 5 per group). Time 0 represents the baseline value before surgery. ***P < 0.001 and *P < 0.05 indicates a statistically significant difference when compared with the sham group. ^#^*P* < 0.01 indicates a statistically significant difference when compared with the CCI group.

**Figure 3:**
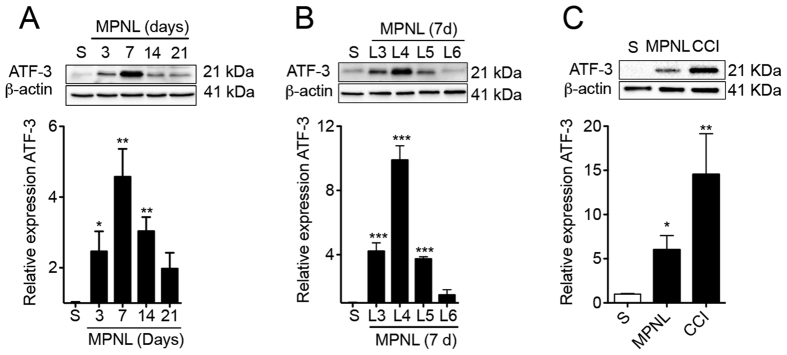
MPNL induces peripheral sensory neuron injury. (**A**) The DRGs L3, L4 and L5 were collected at 3–21 days after MPNL surgery. (**B**) On the seventh day after MPNL, the DRG were collected separately at L3, L4, L5 and L6. (**C**) On the seventh day after MPNL or CCI, the DRG were collected (pooled L3, L4, L5 and L6). Representative blots of the three experiments are shown as well densitometric analyses. The data are presented as the mean ± S.E.M (n = 4 per group). ***P < 0.001, **P < 0.01 and *P < 0.05 indicate statistically significant differences when compared with sham mice.

**Figure 4:**
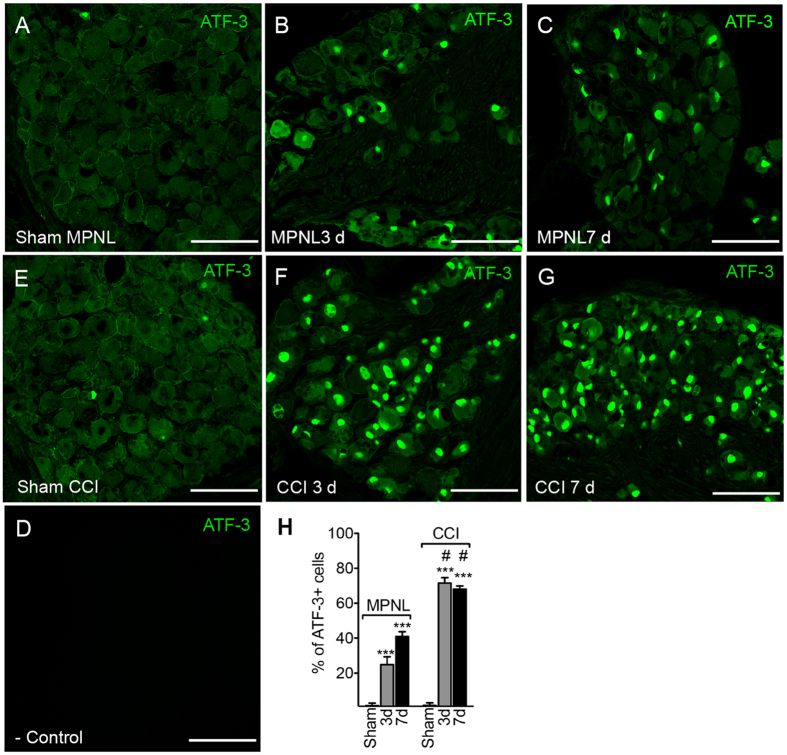
ATF-3 immunoreactivity in the L4 DRG after MPNL or CCI. At 3 or 7 days after MPNL or CCI surgery, L4 DRGs were harvested and the expression of ATF-3 was evaluated by immunofluorescence. ATF-3 was detected exclusively in the nuclei of neurons in the DRG. Representative images are shown in (**A–F**). (**G**) As a control, the primary antibody was omitted from the reaction. Scale bar 50 μm. (**H**) The percentages of ATF-3 + neurons are also shown. The data are presented as the mean ± S.E.M (n = 4 per group). ***P < 0.001 compared with naive mice. ^#^*P* < 0.001 compared with MPNL mice.

**Figure 5:**
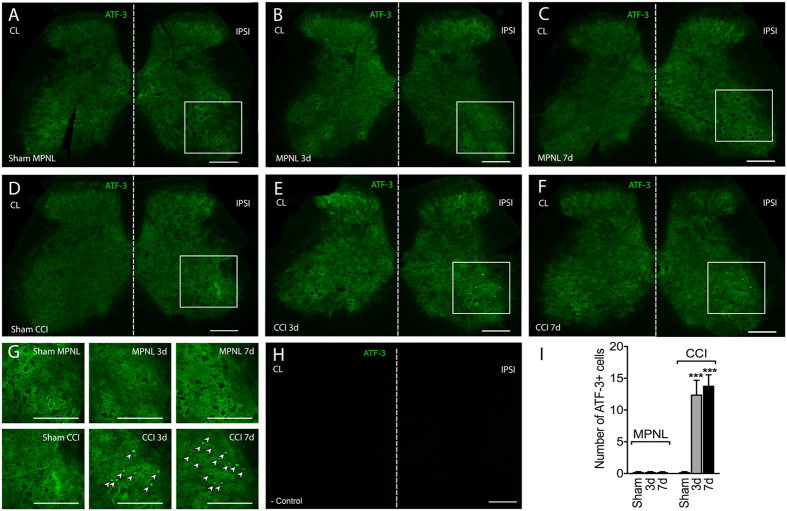
ATF-3 immunoreactivity in the spinal cord after MPNL or CCI. At 3 or 7 days after MPNL or CCI surgery, spinal cords were harvested and the expression of ATF-3 was evaluated by immunofluorescence. ATF-3 was detected exclusively in the nuclei of the ventral horn of the spinal cord of CCI mice. Representative images are shown in (**A–F**). (**G**) ATF-3 + neurons visualized (indicated by arrows) in the dorsal horn of the spinal cord are also shown at a higher magnification. (**H**) As a control, the primary antibody was omitted from the reaction. Scale bar 250 μ m. (**I**) The number of ATF-3 + neurons are also shown. The data are presented as the mean ± S.E.M (n = 4 per group). ***P < 0.001 compared with naive mice. ^#^*P* < 0.001 compared with MPNL mice.

**Figure 6:**
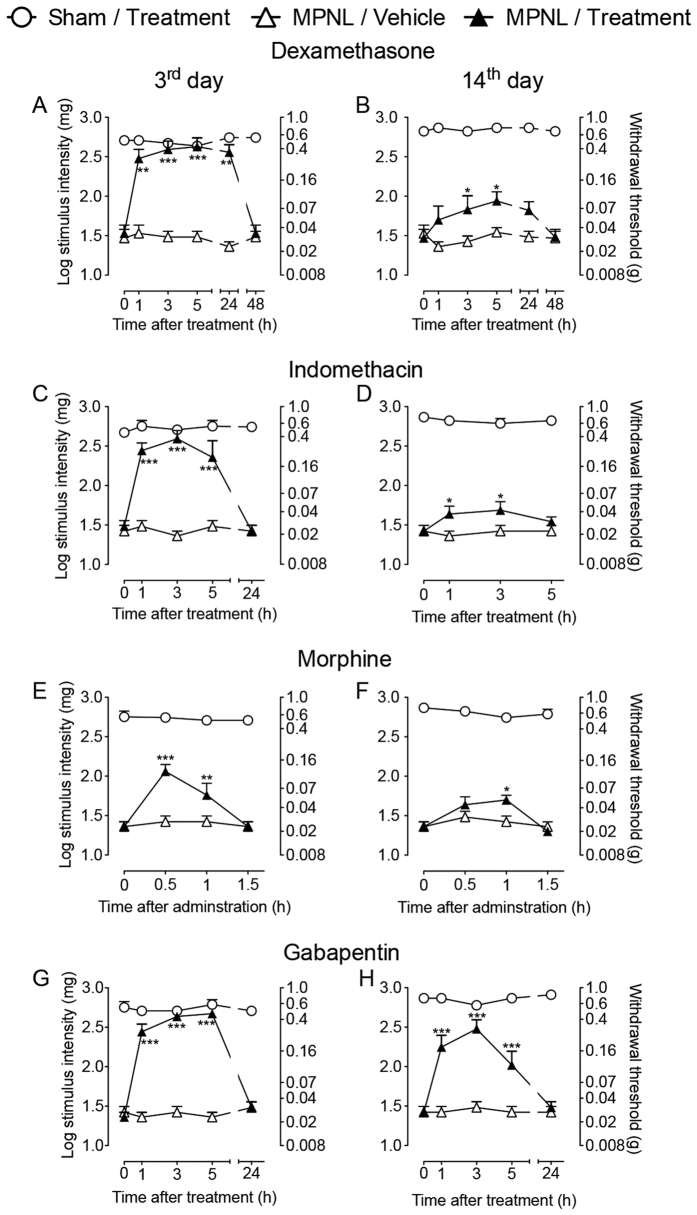
Pharmacological predictivity of pain-like behaviours after MPNL. At 3 or 14 days after MPNL, mice were treated systemically with (**A,B**) dexamethasone (2 mg/kg; s.c), (**C,D**) indomethacin (5 mg/kg, i.p), (**E,F**) morphine (5 mg/kg, i.p) or (**G,H**) gabapentin (100 mg/kg). The withdrawal threshold was measured using von Frey filaments after treatment. The data are presented as the mean ± S.E.M. (n = 5 per group). Time 0 represents the baseline before pharmacological treatments. ***P < 0.001 and **P < 0.01 to *P < 0.05 indicates a statistically significant difference when compared with the vehicle treated group.

**Figure 7:**
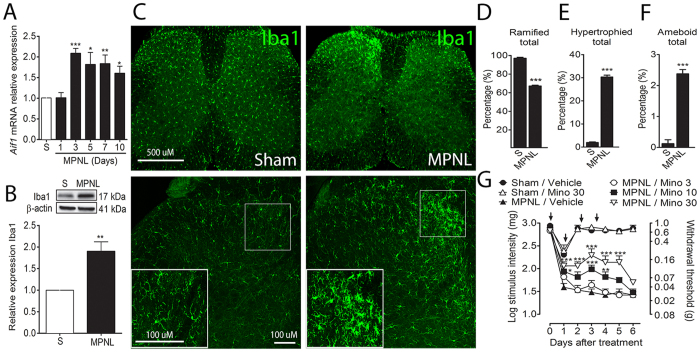
MPNL-induced pain-like behaviours depend on microglial cell activation. The lumbar spinal cord samples were collected at indicated times after MPNL. (**A**) The mRNA for *Aif1* (Iba1) and protein expressions were measured by RT-PCR (n = 6 per group), (**B**) western blotting (representative blotting and analyses of n = 4 per group) or (**C**) immunofluorescence (representative image in different magnifications, n = 4 per group). Scale bars of 500 and 100 μm. (**D–F**). The morphological subtypes of microglial cells (**D**) ramified, (**E**) hypertrophied and (**F**) amoeboid were quantified. (**D**) Mice (n = 5 per group) were treated with minocycline (i.t., 3–30 nmol/site) or vehicle before and 3 days after MPNL. The withdrawal threshold was assessed daily after surgery (3 h after drug administration). Time 0 represents the baseline value before surgery. The data are presented as the mean ± S.E.M. ***P < 0.001 and **P < 0.01 to *P < 0.05 indicates a statistically significant difference when compared with the sham or vehicle treated group.

**Figure 8:**
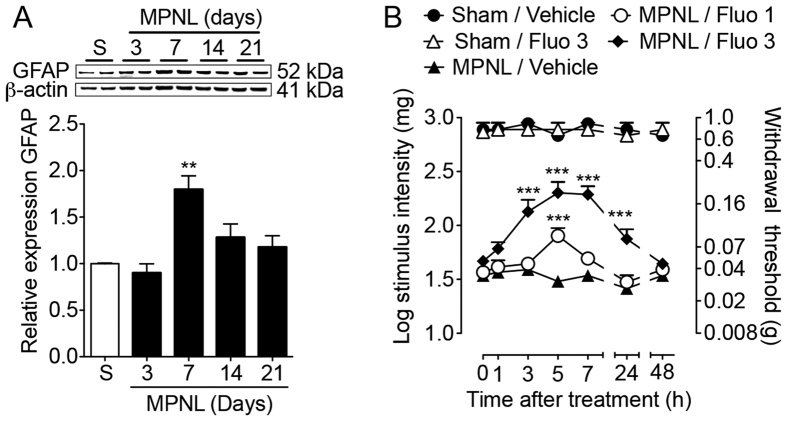
MPNL-induced pain-like behaviours depend on astrocyte activation. (**A**) The lumbar spinal cord samples were collected at indicated times after MPNL or sham. The GFAP protein expression was measured using western blotting (representative blotting and analyses; n = 4 per group). (**B**) The mice (n = 5 per group) were treated with fluorocitrate (i.t., 1–3 nmol/site) 7 days after MPNL, and the withdrawal threshold was assessed before and up to 48 h after fluorocitrate treatment. The data are presented as the mean ± S.E.M. ***P < 0.001 and **P < 0.01 indicates a statistically significant difference when compared with the sham or vehicle treated group.

**Figure 9:**
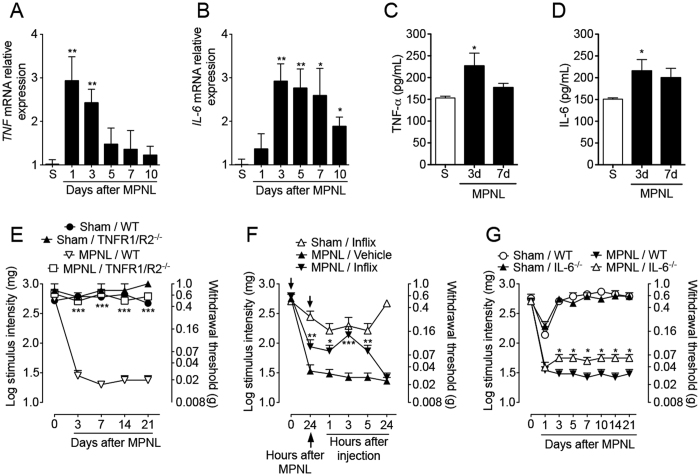
TNF and IL-6 mediate MPNL-induced pain-like behaviours. The lumbar spinal cord samples were collected at indicated times after MPNL. The mRNA or protein for (**A,C**) Tnf and (**B,D**) Il-6 were assessed by RT-PCR and ELISA, respectively. The withdrawal threshold response to mechanical stimulation was assessed in (**C**) WT or TNFR1/2^−/−^ mice up to 21 days after MPNL surgery. (**E**) The mice were treated with infliximab (i.t., 100 μg/site) or vehicle before and 24 h after MPNL. The mechanical withdrawal threshold was assessed 24 after MPNL and up to 24 h after the second dose of infliximab. (**F**) The withdrawal threshold response to mechanical stimulation was assessed in (**C**) IL-6^−/−^ mice up to 21 days after surgery. The results are expressed as the mean ± S.E.M. (n = 5 per group). Time 0 represents the baseline value before surgery. ***P < 0.001 and **P < 0.01 to *P < 0.05 indicates a statistically significant difference when compared with the control groups.

Under isoflurane anaesthesia (1.5% in oxygen), the skin on the medial surface of the ankle was incised (0.5 cm) to expose the medial plantar nerve. After dissection, one ligation of this nerve was performed with 4–0 catgut suture (Ethicon, São Paulo, Brazil). The ligation stayed tightly bound to the nerve without throttling due to the feature of the wire used in the process. Then, 4–0 silk suture was used to join the two edges of the incision (Fig. 1). The sham (control) group procedure involved an incision of approximately 0.5 cm in the medial ankle region of the mice, exposing the medial plantar nerve. However, in this group, the medial plantar nerve was not manipulated.

#### CCI model

Mice were anaesthetized with isoflurane (1.5% in oxygen) followed by trichotomy in the surgical area. CCI was created as previously described^10^. Briefly, the right sciatic nerve was exposed at the mid-thigh level, and four ligatures with 4–0 catgut suture (Ethicon, São Paulo, Brazil) were loosely tied around the nerve just proximal to the trifurcation. For the sham-operation controls, the mice underwent the same procedure without constriction of the nerve.

#### Compounds

The following compounds were used in this study: minocycline (Sigma, #M9511, St. Louis, MO, USA), fluorocitrate (Sigma, #F9634 St. Louis, MO, USA), indomethacin (Sigma, #I7378, St. Louis, MO, USA), dexamethasone (Sigma, #D1159, St. Louis, MO, USA), gabapentin (Tocris Bioscience, #0806, Bristol, United Kingdom), morphine (Cristália, São Paulo, Brazil), and infliximab (Schering-Plough, São Paulo, Brazil).

#### Drug Administration

Intraperitoneal (i.p.): the drugs were injected into the ventral portion of the mouse close to the abdominal midline using a 1 mL syringe with a needle length of 2.5 cm and a gauge of 0.7 cm.

Intrathecal (i.t.): under isoflurane anaesthesia (1.5% in oxygen), trichotomy was performed in the mice dorsal region. Using a 30 unit insulin syringe (BD Ultra Fine TM II), the drugs were administered (5 μL) between the L4 and L5 vertebrae. The animal’s tail reflex findings were expected to confirm the correct performance of the procedure^54^.

#### Nociceptive Test

Animals were placed on an elevated wire grid, and the plantar surfaces of their hind paws were stimulated with a series of ascending force von Frey monofilaments. Mice were first habituated to the experimental environment (room and apparatus) for a period of at least 30 minutes. Mechanical allodynia was accessed by measuring the paw withdrawal threshold taken as the lowest force that evoked a brisk withdrawal response to one of five repetitive stimuli. Briefly, a logarithmic series of 10 calibrated monofilaments (von Frey hairs) was applied to the right hind paws to determine the stimulus intensity threshold stiffness required to elicit a paw withdrawal response. The log stiffness of the hairs is determined by log10 (milligrams) and ranged from 0.903 (8 mg or 0.008 g) to 3.0 (1000 mg or 1 g). The mechanical threshold is represented as previously described^55^,^56^. An investigator who was blinded to the group allocation performed all of the behaviour experiments.

#### Rota-rod Test

The rotarod test was performed to check each animal’s motor coordination. The animals were placed on a rotating roller (20 rpm, initially). After 1 min, the roll speed was increased to a maximum of 36 rpm within 90 seconds. The residence time of each animal in the apparatus was measured by a maximum of 2 minutes. The test was performed three times for each animal with intervals of 20 minutes. The animals were acclimated one day before the experiment.

#### Western Blotting

The dissection and collection of spinal cord tissue from a region that included lumbar segments (L3–L6) and peripheral dorsal root ganglia (segments L3, L4, L5 and L6) ipsilateral to the nerve injury were performed. The tissue was homogenized and lysed in RIPA buffer (*Sigma*, #R0278, St. Louis, MO, USA) and protease inhibitor (Protease Inhibitor Cocktail Tablets - Roche Diagnostics, #04693116001, São Paulo, Brazil). The homogenate was centrifuged at 15,000 g for 10 minutes at 4 °C. An aliquot of the supernatant was separated for the determination of the protein concentration using the Coomassie colorimetric method (Bradford Reagent – Thermo Scientific, Rockford, IL, USA).

The protein samples were separated by electrophoresis on an SDS-PAGE acrylamide gel (gradient 4% to 20%) and transferred to nitrocellulose membranes. The incubation consisted of one hour at room temperature with blocking solution containing 5% blocking agent (GE Healthcare Life Science, Pittsburgh, PA, USA) dissolved in Tris-Buffered Saline - 10% Tween 20 (TBS-T), followed by incubation with primary antibodies anti-GFAP, 1:30000 (Merck Millipore, # AB5541, Darmstadt, Germany), anti-Iba1, 1:400 (*Wako Chemicals, #* 019–19741, Osaka, Japan) and anti-ATF-3, 1:400 (*Santa Cruz Biotechnology*, # sc-188, CA, USA) overnight at 4 °C in TBS-T/5% bovine serum albumin (BSA). Then, the membranes were washed in TBS-T 3 times over 5 minutes and were incubated for one hour with secondary antibodies conjugated with peroxidase at 1:5000 in 5% blocking agent (GE Healthcare Life Science, # RPN2125, São Paulo, Brazil) dissolved in TBS-T. The membranes were incubated with HRP Substrate Luminata^TM^ Fort Western (*Millipore*, # WBLUF0500, São Paulo, Brazil) and were then revealed, as indicated by the manufacturer. Image capture was performed with a Quemi TM-Doc XRS apparatus as directed by the manufacturer. The densitometric data were measured following normalization to the control using Scientific Imaging Systems (Image labTM 3.0 software, Biorad Laboratories, Hercules CA).

#### Real-time RT-PCR

After collection of tissue from the region that included the lumbar segments (L4–L6) of the spinal cord ipsilateral to the lesion, tissues were rapidly homogenized in TRIzol reagent (Invitrogen Life Technologies Corporation, Carlsbad, California, USA), and 0.2 mL of chloroform was added to the samples, which were shaken. The suspension was centrifuged for 13000 *g* at 4 °C for 15 min. The aqueous phase was transferred to a fresh tube to which 0.75 mL of isopropanol was added. After mixing, the samples were incubated for 2 hours at −20 °C. The samples were centrifuged for 15 min at 13000 *g* (4 °C). The RNA precipitate was washed with 0.5 mL of ethanol, and the preparation was suspended in 20 μL of PCR-grade H_2_O. RNA concentrations were determined by optical density at a wavelength of 260 nm with the NanoVue ® apparatus (*GE Healthcare*). One microgram of total RNA was transcribed into cDNA by reverse transcriptase enzyme action Improm Pre-II ® (Promega, Madison, Wisconsin, USA). Quantitative RT-PCR in real time was performed on an ABI Prism ® 7500 Sequence Detection System (*Applied Biosystems*) using SYBR-green System Fluorescence (*Applied Biosystems, Warrington, UK*) for quantification during amplification. RT-PCR was performed with a final reaction volume of 6.25 μL; RT-PCR was initially kept at 95 °C (10 min), followed by 40 cycles of 94 °C (1 min), 56 °C (1 min) and 72 °C (2 min). The melting curve was analysed (65–95 °C) to verify that only one product was amplified. Samples with more than one peak were excluded. The results were analysed using the method of quantitative relative expression 2^−ΔΔCt ^57.

The primer sequences were as follows in Table 1:

#### Immunofluorescence staining and analyses

The animals were deeply anesthetized with ketamine and xylazine (2:1) and perfused through the ascending aorta with PBS, followed by 4% paraformaldehyde. Spinal cord sections (60 μm, free-floating technic) and DRG sections (10 μm, one the glass technic) were obtained using a cryostat. The spinal cord sections were washed in PBS (0.01 M, pH 7.4) 3 times over 5 minutes and incubated in 5% normal goat serum and 1% BSA dissolved in PBS with 0.1% Triton X100 (PBS-T) for 1 hour. Subsequently, the sections were washed in PBS-T (0.01 M, pH 7.4) 3 times over 5 minutes and then subjected to immunofluorescence staining with overnight incubation at 4 °C with polyclonal anti-Iba1 (1:1000; Wako Chemicals, Osaka, Japan) or rabbit polyclonal anti-ATF3 (1:500; Santa Cruz Biotechnology, # sc-188, CA, USA). After incubation with the primary antibodies, the sections were washed in PBS-T 3 times over 5 minutes and incubated at room temperature for 2 hours with Alexa Fluor 488^®^ goat anti-rabbit IgG (*Molecular Probes*, Molecular Probes, Carlsbad, CA, USA). The sections were washed with PBS-T as described earlier, mounted on glass slides with Fluoromount^TM^ Aqueous Mounting Medium (*Sigma*, St. Louis, MO, USA), and then covered with cover slips. The DRG sections were fixed on slides and subsequently immersed in TBS and 50%, 70% and 50% ethanol solutions for 10 minutes each to increase the permeability. Then, the slides were washed in TBS 3 times over 10 minutes, and the blocking step was performed with 5% normal goat serum (NGS) in TBS for 60 minutes at room temperature. Subsequently, the slides were subjected to immunofluorescence staining with an overnight primary antibody incubation at 4 °C with rabbit polyclonal anti-ATF3 (1:500, Santa Cruz Biotechnology, # sc-188, CA, USA) antibody diluted in 5% NGS. As a control, primary antibodies were omitted from the reaction. They were washed in TBS 4 times over 5 min, incubated at room temperature for 60 min with Alexa Fluor 488^®^ goat anti-rabbit IgG (Molecular Probes, Carlsbad, CA, USA) diluted in 5% NGS in TBS. The slides were washed in TBS 4 times over 5 minutes and further covered with a coverslip as described above. A confocal microscope (SP5, Leica, Wetzlar, Germany) was used to visualize and capture Iba1 and ATF-3 staining sections in the DRG and spinal cord under the same light conditions. For the quantitative analysis, at least five slices from the spinal cords and at least five from the DRGs from each mouse were used for the Iba1 and ATF-3 immunoreactive quantification. The percentage of ATF3-labeled neurons in the DRGs was calculated by dividing the number of ATF3+ neurons by the total number of neurons counted× 100. In the spinal cord, the number of ATF-3+ neurons per slices was quantified.

Microglia were classified as previously described^58^. In brief, ramified microglia were classified as having a normal pattern, with fine, mild and radially projecting processes. Hypertrophied microglia were defined as having an enlarged and dimmed cell body with denser, shorter and lesser projecting processes. Finally, amoeboid microglia were defined as densely stained and having enlarged soma with very few or no processes, which was usually accompanied with the presence of filopodia. The percentage of a specific morphological type was accessed by calculating the ratio of the total cell type morphology to the total number of microglial cells observed in the field.

#### Cytokine measurement

Mice were terminally anaesthetized with ketamine and xylazine (2:1) and perfused through the ascending aorta with PBS at specific time points after MPNL induction. After the collection of tissue from the region including lumbar segments (L4–L6) of the spinal cord ipsilateral to the lesion, these tissues were rapidly homogenized in 150 μl of buffer containing protease inhibitors. TNFα and IL-6 concentrations were determined by ELISA using commercial kits (DuoSet; R&D Systems, Minneapolis, MN, USA) and paired antibodies as instructed by the manufacturer.

#### Data analysis and statistics

All the results are presented as the means ± S.E.M. For the behaviour tests, we used 5 mice/group, and the results are representative of 2 independent experiments. For western blotting, immunofluorescence and ELISA assays, we used 4 mice/group, whereas for the PCR assays, we used 6 mice/group. Two-way analysis of variance (ANOVA) was used to compare groups and doses at all time points (curves) when the nociceptive responses were measured at different time points after the stimulus injection. The factors analysed were the treatments, time and time by treatment interactions. When there was a significant time by treatment interaction; one-way ANOVA followed by Bonferroni’s *t* test was performed for each time point. Alternatively, when the hypernociceptive responses were measured once after the stimulus injection, the differences between responses were evaluated by one-way ANOVA followed by *Tukey’s t* test. *P* < 0.05 was considered as significant.

## Additional Information

**How to cite this article**: Sant’Anna, M. B. *et al.* Medial plantar nerve ligation as a novel model of neuropathic pain in mice: pharmacological and molecular characterization. *Sci. Rep.* **6**, 26955; doi: 10.1038/srep26955 (2016).

## Figures and Tables

**Table 1:** Primers used for real-time PCR.

Gene name	Forward sequence	Reverse sequence
*GAPDH*	5′-GGGTGTGAACCACGAAGAAAT	5′-CCTTCCACAATGCCAAAGTT
*TNF-α*	5′-AGGGATGAGAAGTTCCCAATG	5′-GGCTTGTCACTCGAATTTTGAGA
*IL-6*	5′-TTCCTACCCCAATTTCCAAT	5′-CCTTCTGTACTCCCAGCTTATC
*GFAP*	5′-AGGGCGAAGAAAACCGCATCACC	5′-TCTAAGGGAGAGCTGGCAGGGCT
*Aif1*	5′-TGAGGAGCCATGAGCCAAAG	5′-GCTTCAAGTTTGGACGGCAG

## References

[b1] Torrance N., Smith B. H., Watson M. C. & Bennett M. I. Medication and treatment use in primary care patients with chronic pain of predominantly neuropathic origin. Fam. Pract. 24, 481–5 (2007). 17670804 10.1093/fampra/cmm042

[b2] O’Connor A. B. & Dworkin R. H. Treatment of neuropathic pain: an overview of recent guidelines. Am. J. Med. 122, S22–32 (2009). 19801049 10.1016/j.amjmed.2009.04.007

[b3] Costigan M., Scholz J. & Woolf C. J. Neuropathic Pain: A Maladaptive Response of the Nervous System to Damage Michael. Annu Rev Neurosci 32, 1–32 (2010). 19400724 10.1146/annurev.neuro.051508.135531 PMC2768555

[b4] Campbell J. N. & Meyer R. a. Mechanisms of neuropathic pain. Neuron 52, 77–92 (2006). 17015228 10.1016/j.neuron.2006.09.021 PMC1810425

[b5] Berge O.-G. Predictive validity of behavioural animal models for chronic pain. Br. J. Pharmacol. 164, 1195–206 (2011). 21371010 10.1111/j.1476-5381.2011.01300.x PMC3229757

[b6] Zhang H., Zhang H. & Dougherty P. M. Dynamic effects of TNF-α on synaptic transmission in mice over time following sciatic nerve chronic constriction injury. J. Neurophysiol. 110, 1663–71 (2013). 23864372 10.1152/jn.01088.2012 PMC4042422

[b7] Marinelli S. *et al.* Botulinum neurotoxin type A counteracts neuropathic pain and facilitates functional recovery after peripheral nerve injury in animal models. Neuroscience 171, 316–328 (2010). 20826198 10.1016/j.neuroscience.2010.08.067

[b8] Mika J. *et al.* Differential activation of spinal microglial and astroglial cells in a mouse model of peripheral neuropathic pain. Eur. J. Pharmacol. 623, 65–72 (2009). 19766105 10.1016/j.ejphar.2009.09.030

[b9] Pinheiro F. D. V. *et al.* The involvement of the TRPA1 receptor in a mouse model of sympathetically maintained neuropathic pain. Eur. J. Pharmacol. 747, 105–113 (2015). 25498793 10.1016/j.ejphar.2014.11.039

[b10] Bennett G. J. & Xie Y. K. A peripheral mononeuropathy in rat that produces disorders of pain sensation like those seen in man. Pain 33, 87–107 (1988). 2837713 10.1016/0304-3959(88)90209-6

[b11] Ji R.-R. & Suter M. R. p38 MAPK, microglial signaling, and neuropathic pain. Mol. Pain 3, 33 (2007). 17974036 10.1186/1744-8069-3-33 PMC2186318

[b12] Beggs S. & Salter M. W. The known knowns of microglia-neuronal signalling in neuropathic pain. Neurosci. Lett. 557, 37–42 (2013). 23994389 10.1016/j.neulet.2013.08.037

[b13] Mika J., Zychowska M., Popiolek-Barczyk K., Rojewska E. & Przewlocka B. Importance of glial activation in neuropathic pain. Eur. J. Pharmacol. 716, 106–19 (2013). 23500198 10.1016/j.ejphar.2013.01.072

[b14] Tsuda M., Beggs S., Salter M. W. & Inoue K. Microglia and intractable chronic pain. Glia 61, 55–61 (2013). 22740331 10.1002/glia.22379

[b15] Uçeyler N. & Sommer C. Cytokine regulation in animal models of neuropathic pain and in human diseases. Neurosci. Lett. 437, 194–198 (2008). 18403115 10.1016/j.neulet.2008.03.050

[b16] Grace P. M., Hutchinson M. R., Maier S. F. & Watkins L. R. Pathological pain and the neuroimmune interface. Nat. Rev. Immunol. 1–15 (2014). 24577438 10.1038/nri3621 PMC5525062

[b17] De-la-Llave-Rincón A. I., Puentedura E. J. & Fernández-de-las-Peñas C. New advances in the mechanisms and etiology of carpal tunnel syndrome. Discov. Med. 13, 343–8 (2012). 22642915

[b18] Challa S. R. Surgical animal models of neuropathic pain: Pros and Cons. Int. J. Neurosci. 125, 170–4 (2015). 24831263 10.3109/00207454.2014.922559

[b19] Gupta R. & Steward O. Chronic Nerve Compression Induces Concurrent Apoptosis and Proliferationof Schwann Cells. J. Comp. Neurol. 461, 174–186 (2003). 12724836 10.1002/cne.10692

[b20] Schmid A. B., Coppieters M. W., Ruitenberg M. J. & McLachlan E. M. Local and Remote Immune-Mediated Inflammation After Mild Peripheral Nerve Compression in Rats. J. Neuropathol. Exp. Neurol. 72, 662–680 (2013). 23771220 10.1097/NEN.0b013e318298de5b

[b21] Schmid D., Messlinger K., Belmonte C. & Fischer M. J. M. Altered thermal sensitivity in neurons injured by infraorbital nerve lesion. Neurosci. Lett. 488, 168–172 (2011). 21078368 10.1016/j.neulet.2010.11.022

[b22] Dworlzin R., Allen R. & Saltorelli M. Advances in Neuropathic Pain: Diagnosis, Mechanisms, and Treatment Recommendations Robert. Arch. Neurol. 60, 1524–34 (2003). 14623723 10.1001/archneur.60.11.1524

[b23] Woolf C. J. & Mannion R. J. Neuropathic pain: aetiology, symptoms, mechanisms, and management. Lancet 353, 1959–64 (1999). 10371588 10.1016/S0140-6736(99)01307-0

[b24] Hai T., Wolfgang C. D., Marsee D. K., Allen A. E. & Sivaprasad U. ATF3 and stress responses. In *Gene Expr*. 7, 321–335 (1999). PMC6174666 10440233

[b25] Tsujino H. *et al.* Activating transcription factor 3 (ATF3) induction by axotomy in sensory and motoneurons: A novel neuronal marker of nerve injury. Mol. Cell. Neurosci. 15, 170–82 (2000). 10673325 10.1006/mcne.1999.0814

[b26] Thakur M., Rahman W., Hobbs C., Dickenson A. H. & Bennett D. L. H. Characterisation of a peripheral neuropathic component of the rat monoiodoacetate model of osteoarthritis. PLos one 7, e33730 (2012). 22470467 10.1371/journal.pone.0033730 PMC3312347

[b27] Obata K., Yamanaka H., Fukuoka T., Yi D. & Tokunaga A. Contribution of injured and uninjured dorsal root ganglion neurons to pain behavior and the changes in gene expression following chronic constriction injury of the sciatic nerve in rats. Pain 101, 65–77 (2003). 12507701 10.1016/s0304-3959(02)00296-8

[b28] Loram L. C. *et al.* Systemic administration of an alpha-7 nicotinic acetylcholine agonist reverses neuropathic pain in male Sprague Dawley rats. J. pain 13, 1162–71 (2012). 23182225 10.1016/j.jpain.2012.08.009 PMC5654381

[b29] Finnerup N. B. *et al.* Pharmacotherapy for neuropathic pain in adults: a systematic review and meta-analysis. Lancet 14, 162–173 (2015). 25575710 10.1016/S1474-4422(14)70251-0 PMC4493167

[b30] Moore A., Wiffen P. & Kalso E. Antiepileptic drugs for neuropathic pain and fibromyalgia. JAMA 312, 182–3 (2014). 25005656 10.1001/jama.2014.6336

[b31] Yasuda T., Miki S., Yoshinaga N. & Senba E. Effects of amitriptyline and gabapentin on bilateral hyperalgesia observed in an animal model of unilateral axotomy. Pain 115, 161–70 (2005). 15836979 10.1016/j.pain.2005.02.026

[b32] Inoue N. *et al.* Full Paper Etodolac Attenuates Mechanical Allodynia in a Mouse Model of Neuropathic Pain. J. Pharmacol. Sci. 605, 600–605 (2009). 19346674 10.1254/jphs.08287fp

[b33] Besson J. M. The neurobiology of pain. Lancet 353, 1610–5 (1999). 10334274 10.1016/s0140-6736(99)01313-6

[b34] Millan M. J. The induction of pain: an integrative review. Prog. Neurobiol. 57, 1–164 (1999). 9987804 10.1016/s0301-0082(98)00048-3

[b35] Zanette G., Cacciatori C. & Tamburin S. Central sensitization in carpal tunnel syndrome with extraterritorial spread of sensory symptoms. Pain 148, 227–236 (2010). 20004060 10.1016/j.pain.2009.10.025

[b36] Coderre T. J., Katz J., Vaccarino a. L. & Melzack R. Contribution of central neuroplasticity to pathological pain: Review of clinical and experimental evidence. Pain 52, 259–285 (1993). 7681556 10.1016/0304-3959(93)90161-H

[b37] Basbaum A., Bautista D., Scherrer G. & Julius D. Cellular and molecular mechanisms of pain. Cell (2009). 10.1016/j.cell.2009.09.028 PMC2852643 19837031

[b38] Gold M. S. & Gebhart G. F. Nociceptor sensitization in pain pathogenesis. Nat. Med. 16, 1248–1257 (2010). 20948530 10.1038/nm.2235 PMC5022111

[b39] Ji R.-R., Berta T. & Nedergaard M. Glia and pain: is chronic pain a gliopathy? Pain 154 Suppl,S10–28 (2013). 23792284 10.1016/j.pain.2013.06.022 PMC3858488

[b40] Milligan E. D. & Watkins L. R. Pathological and protective roles of glia in chronic pain. Nat Rev Neurosci. 10, 23–36 (2009). 19096368 10.1038/nrn2533 PMC2752436

[b41] Jha M. K., Jeon S. & Suk K. Glia as a Link between Neuroinflammation and Neuropathic Pain. Immune Netw. 12, 41–7 (2012). 22740789 10.4110/in.2012.12.2.41 PMC3382663

[b42] Ledeboer A. *et al.* Minocycline attenuates mechanical allodynia and proinflammatory cytokine expression in rat models of pain facilitation. Pain 115, 71–83 (2005). 15836971 10.1016/j.pain.2005.02.009

[b43] Mika J., Osikowicz M., Makuch W. & Przewlocka B. Minocycline and pentoxifylline attenuate allodynia and hyperalgesia and potentiate the effects of morphine in rat and mouse models of neuropathic pain. Eur. J. Pharmacol. 560, 142–9 (2007). 17307159 10.1016/j.ejphar.2007.01.013

[b44] Calvo M., Dawes J. M. & Bennett D. L. H. The role of the immune system in the generation of neuropathic pain. Lancet Neurol. 11, 629–42 (2012). 22710756 10.1016/S1474-4422(12)70134-5

[b45] Milligan E. D. & Watkins L. R. Pathological and protective roles of glia in chronic pain. Nat. Rev. Neurosci. 10, 23–36 (2009). 19096368 10.1038/nrn2533 PMC2752436

[b46] Clark A. K., Old E. A. & Malcangio M. Neuropathic pain and cytokines : current perspectives. J. Pain Res. 803–814 (2013). 24294006 10.2147/JPR.S53660 PMC3839806

[b47] Kobayashi Y., Kiguchi N., Maeda T., Ozaki M. & Kishioka S. The critical role of spinal ceramide in the development of partial sciatic nerve ligation-induced neuropathic pain in mice. Biochem. Biophys. Res. Commun. 421, 318–322 (2012). 22503971 10.1016/j.bbrc.2012.03.153

[b48] Kleinschnitz C., Brinkhoff J., Zelenka M., Sommer C. & Stoll G. The extent of cytokine induction in peripheral nerve lesions depends on the mode of injury and NMDA receptor signaling. J. Neuroimmunol. 149, 77–83 (2004). 15020067 10.1016/j.jneuroim.2003.12.013

[b49] Feng X. *et al.* Intrathecal administration of clonidine attenuates spinal neuroimmune activation in a rat model of neuropathic pain with existing hyperalgesia. Eur. J. Pharmacol. 614, 38–43 (2009). 19397906 10.1016/j.ejphar.2009.04.044

[b50] Inoue K. The function of microglia through purinergic receptors: neuropathic pain and cytokine release. Pharmacol. Ther. 109, 210–26 (2006). 16169595 10.1016/j.pharmthera.2005.07.001

[b51] George A., Buehl A. & Sommer C. Wallerian degeneration after crush injury of rat sciatic nerve increases endo- and epineurial tumor necrosis factor-alpha protein. Neurosci. Lett. 372, 215–219 (2004). 15542243 10.1016/j.neulet.2004.09.075

[b52] Manjavachi M. N., Costa R., Quintão N. L. & Calixto J. B. The role of keratinocyte-derived chemokine (KC) on hyperalgesia caused by peripheral nerve injury in mice. Neuropharmacology 79, 17–27 (2014). 24184386 10.1016/j.neuropharm.2013.10.026

[b53] Oprée A. & Kress M. Involvement of the Proinflammatory Cytokines Tumor Necrosis Factor-α, IL-1β, and IL-6 But Not IL-8 in the Development of Heat Hyperalgesia : Effects on Heat-Evoked Calcitonin Gene-Related Peptide Release from Rat Skin. J. Neurosci. 20, 6289–6293 (2000). 10934280 10.1523/JNEUROSCI.20-16-06289.2000 PMC6772609

[b54] Hylden J. L. K. & Wilcox G. L. Intrathecal morphine in mice: A new technique. Eur. J. Pharmacol. 67, 313–316 (1980). 6893963 10.1016/0014-2999(80)90515-4

[b55] Tal M. & Bennett G. J. Extra-territorial pain in rats with a peripheral mononeuropathy: mechano-hyperalgesia and mechano-allodynia in the territory of an uninjured nerve. Pain 57, 375–82 (1994). 7936715 10.1016/0304-3959(94)90013-2

[b56] Hains L. E. *et al.* Pain intensity and duration can be enhanced by prior challenge: Initial evidence suggestive of a role of microglial priming. J. Pain 11, 1004–1014 (2010). 20434956 10.1016/j.jpain.2010.01.271 PMC2916950

[b57] Livak K. J. & Schmittgen T. D. Analysis of relative gene expression data using real-time quantitative PCR and the 2(-Delta Delta C(T)) Method. Methods 25, 402–408 (2001). 11846609 10.1006/meth.2001.1262

[b58] Ayoub A. E. & Salm a K. Increased morphological diversity of microglia in the activated hypothalamic supraoptic nucleus. J. Neurosci. 23, 7759–7766 (2003). 12944504 10.1523/JNEUROSCI.23-21-07759.2003 PMC6740605

